# Comprehensive metabolic characterization of serum osteocalcin action in a large non-diabetic sample

**DOI:** 10.1371/journal.pone.0184721

**Published:** 2017-09-18

**Authors:** Lukas Entenmann, Maik Pietzner, Anna Artati, Anke Hannemann, Ann-Kristin Henning, Gabi Kastenmüller, Henry Völzke, Matthias Nauck, Jerzy Adamski, Henri Wallaschofski, Nele Friedrich

**Affiliations:** 1 Institute of Clinical Chemistry and Laboratory Medicine, University Medicine Greifswald, Greifswald, Germany; 2 Institute of Experimental Genetics, Genome Analysis Center, Helmholtz Zentrum München, Neuherberg, Germany; 3 Institute of Bioinformatics and Systems Biology, Helmholtz Zentrum München, German Research Center for Environmental Health, Neuherberg, Germany; 4 Institute for Community Medicine, University Medicine Greifswald, Greifswald, Germany; 5 DZHK (German Center for Cardiovascular Research), partner site Greifswald, Greifswald, Germany; 6 Lehrstuhl für Experimentelle Genetik, Technische Universität München, Freising-Weihenstephan, Germany; 7 German Center for Diabetes Research (DZD), München-Neuherberg, Germany; 8 Schwerpunktpraxis für Diabetes und Hormonerkrankungen, Erfurt, Germany; 9 Research Centre for Prevention and Health, Capital Region of Denmark, Glostrup, Denmark; University of Alabama at Birmingham, UNITED STATES

## Abstract

Recent research suggested a metabolic implication of osteocalcin (OCN) in e.g. insulin sensitivity or steroid production. We used an untargeted metabolomics approach by analyzing plasma and urine samples of 931 participants using mass spectrometry to reveal further metabolic actions of OCN. Several detected relations between OCN and metabolites were strongly linked to renal function, however, a number of associations remained significant after adjustment for renal function. Intermediates of proline catabolism were associated with OCN reflecting the implication in bone metabolism. The association to kynurenine points towards a pro-inflammatory state with increasing OCN. Inverse relations with intermediates of branch-chained amino acid metabolism suggest a link to energy metabolism. Finally, urinary surrogate markers of smoking highlight its adverse effect on OCN metabolism. In conclusion, the present study provides a read-out of metabolic actions of OCN. However, most of the associations were weak arguing for a limited role of OCN in whole-body metabolism.

## Introduction

Osteocalcin (OCN) is a γ-carboxylated protein of 49 amino acids produced by osteoblasts, odontoblasts and hypertrophic chondrocytes. It has a central role in the bone remodeling cycle, affecting bone mineralization, and is considered as a marker of bone formation, but its function is not yet entirely understood [1]. OCN is released from the bone matrix into the blood stream following matrix resorption [2] and is freely filtered by the kidneys. Fully γ-carboxylated OCN is able to bind calcium and in consequence hydroxylapatite in bone. Next to the fully γ-carboxylated form, OCN also exists in forms with reduced amount of carboxylated glutamate residues.

Apart from bone metabolism, circulating OCN seems to act in a hormone-like manner. OCN deficient mice showed decreased glucose tolerance and accumulation of body fat [3]. Furthermore, *in vitro* and *in vivo* experiments revealed that both the fully carboxylated and the undercarboxylated OCN, stimulate glucose transport and oxidation as well as insulin sensitivity in myocytes and adipocytes [3]. A similar effect, namely OCN being a powerful synergist in management of insulin synthesis and release, was observed in a pharmacological intervention study in mice treated with OCN combined with a high-fat diet [4]. On the other side, insulin seems to play an important role in OCN production and bioactivity regulation. Mice lacking insulin receptors in osteoblasts accumulated body fat and developed insulin resistance similar to OCN deficient mice [5]. A feed-forward loop between bone and pancreas was thus suggested [5].

Similar effects were also reported in humans. For instance, a study [6] in elderly men demonstrated strong inverse correlations between plasma OCN concentrations and indicators of altered energy metabolism such as fat mass, body mass index (BMI) or plasma glucose concentration. The serum OCN concentration was further proposed to predict incident type 2 diabetes in middle-aged subjects [7]. Besides the effects on glucose metabolism, an influence of OCN on the reproductive system, including the regulation of the intracellular 25-hydroxy vitamin D concentration in mice Leydig cells [8] and positive associations between serum OCN and serum testosterone concentrations were reported in healthy men as well as in patients suffering from bone diseases [9].

To address possible implications of circulating OCN on human metabolism in a comprehensive manner, techniques like metabolomics can be used [10]. This technique allows to assess the content of small molecules present in biofluids, cells or tissues, which directly reflects the metabolic state of the organism, and to relate the metabolic profile to the serum OCN concentration. We therefore attempted to analyze possible metabolic implications of the serum OCN concentration in humans using state-of-the-art untargeted mass spectrometry (MS)-based plasma and urine metabolome data of a non-diabetic sample of the general population.

## Material and methods

### Study population

The Study of Health in Pomerania (SHIP-TREND) is a population-based study located in West Pomerania, a rural region in northeast Germany [11]. A stratified (age, sex and city/county of residence) random sample of 8826 adults aged 20–79 years was drawn from population registries. Sample selection was facilitated by centralization of local population registries in the Federal State of Mecklenburg-West Pomerania. Baseline examinations were conducted between 2008 and 2012. In total, 4420 subjects chose to participate (50.1% response). All participants gave written informed consent before taking part in the study. The study was approved by the ethics committee of the University of Greifswald and conformed to the principles of the declaration of Helsinki. SHIP data are publically available for scientific and quality control purposes. Data usage can be applied for via www.community-medicine.de to ensure compliance with all legislation.

For a subsample of 995 participants without self-reported diabetes, plasma as well as urine metabolomome data based on MS (see below) were acquired. Participants exhibiting at least one of the following characteristics were excluded from the study sample (overlap exists): missing values in exposure or confounder (n = 13), renal failure (estimated glomerular filtration rate (eGFR) <60 mL/min/1.73m^2^ n = 7), intake of medication influencing the serum OCN concentration (bisphosphonates and combinations, selective estrogen receptor modulators, parathyroid hormones and analogues or calcitonin, corticosteroids and combinations, strontium ranelate, vitamin D and analogues; n = 16), newly diagnosed diabetes (n = 30) or extreme parathyroid hormone concentrations (>120 pg/mL; n = 7). Finally 931 participants, comprising 414 men and 517 women, were included in the present study.

### Laboratory measurements and phenotypic characterization

Smoking status (current, former or never smokers), daily alcohol consumption and physical activity (≥1 h training a week) were assessed using computer-aided personal interviews. Waist circumference (WC) was measured to the nearest 0.1 cm using an inelastic tape midway between the lower rib margin and the iliac crest in the horizontal plane. Hypertension was defined as an increased blood pressure (BP) (systolic BP of ≥140 mmHg or diastolic BP of ≥90 mmHg) or the use of antihypertensive medication. The intake of oral contraceptives (OC; ATC: G03A) or postmenopausal hormone therapy (PHT; ATC: G03C, G03D and G03F, N = 31) were defined based on ATC codes.

Fasting blood samples were taken from the cubital vein of participants in the supine position between 7.00 a.m. and 12.00 p.m. In the same time span spot urine samples were taken. All samples were either analyzed immediately or stored at −80°C. Serum OCN concentrations were measured with the IDS-iSYS N-Mid Osteocalcin assay on the IDS-iSYS Multi-Discipline Automated Analyser (Immunodiagnostic Systems Limited, Frankfurt am Main, Germany) according to the instructions for use. This assay detects the intact OCN polypeptide (amino acids 1–49) and the N-terminal-Mid OCN fragment (amino acids 1–43). The measurement range of the assay was 2–200 ng/mL. The limits of blank and detection were 0.27 ng/mL. The limit of quantitation was 1.57 ng/mL. As recommended by the manufacturer, three levels of control material were measured. During the course of the study, the coefficients of variation were 13.4% at low, 15.0% at medium, and 17.0% at high serum OCN concentrations in the control material. Serum cystatin C concentrations were measured using a nephelometric assay (Dimension VISTA, Siemens Healthcare Diagnostics, Eschborn, Germany) with a functional sensitivity of 0.05 mg/L. The cystatin C-based eGFR was calculated using the CKD-EPI cystatin C equation: eGFR = 133 × min(serum cystatin C / 0.8, 1)^-0.499^ × max(serum cystatin C / 0.8, 1)^-1.328^ × 0.996^age^ [× 0.932 if female] [12].

### Metabolomics measurements

Non-targeted metabolomics analysis for metabolic profiling was conducted at the Genome Analysis Center, Helmholtz Zentrum München, Germany. A detailed description of metabolite measurements, annotations and data processing is given in the appendix (S1 Appendix). Briefly, two separate LC-MS/MS analytical methods were used, as previously published [13], to obtain broad, untargeted plasma and urine metabolite spectra. After preprocessing, 475 plasma and 558 urine metabolites remained for the statistical analyses. Some of these metabolites could not be unambiguously assigned to a chemical identity and are therefore referred to with the notation “X” and a unique number.

### Statistical analysis

For descriptive analyses continuous data were expressed as median (25th; 75th quartile) and nominal data as percentage. For bivariate comparison of men and women, the Mann–Whitney U test (continuous data) and the χ2 test (nominal data) were used. Associations between OCN concentrations as exposure and the plasma and urine metabolome as outcome were tested by linear regression analyses controlling for age, sex, WC and physical activity. Since the first results revealed a high number of significantly associated metabolites, including markers of kidney function, we further adjusted for the eGFR, the intake of OC or PHT as well as smoking behavior in a second model. The association between the serum OCN concentration and the eGFR was tested using partial correlation coefficients, controlling for age, sex and WC. To account for multiple testing, we adjusted the p-values by controlling the false discovery rate (FDR) at 5% using the Benjamini-Hochberg procedure. This type of correction allows restricting the occurrence of false-positive findings among all nominal significant findings to a certain threshold. In our study maximally 5% of the presented findings might be false positives.

## Results

General characteristics of the 414 men and 517 women from the study population are displayed in Table 1. Men were more often smoker or former smoker and had a higher WC as well as eGFR than women. Every fourth women reported intake of OC or PHT. Sex differences in OCN concentrations and bone-associated markers like parathyroid hormone or 25-hydroxy vitamin D were not found.

**Table 1:** General characteristics of the study population.

Characteristic	Men (n = 414)	Women (n = 517)	p*
Age (years)	50 (39; 61)	51 (40; 59)	0.84
Smoking (%)			
never smoker	31.4	50.5	<0.01
former smoker	44.7	28.0	
current smoker	23.9	21.5	
Physically inactive (%)	27.3	27.5	0.95
Waist circumference (cm)	94 (86; 102)	81 (74; 90)	<0.01
Osteocalcin (ng/mL)	17.3 (13.3; 21.8)	17.4 (13.5; 23.2)	0.33
Parathyroid hormone (pg/mL)	31.7 (23.6; 40.9)	31.0 (23.9; 39.1)	0.38
25-hydroxy vitamin D (μg/L)	25.0 (18.2; 32.6)	24.3 (16.6; 32.9)	0.38
eGFR (mL/min/1.73m^2^)	117.6 (108.1; 125.1)	112.9 (103.9; 121.4)	<0.01
Hormone intake (%)**	-	24.4	

eGFR = estimated glomerular filtration rate based on cystatin C. Continuous data are expressed as median (25th percentile; 75th percentile); nominal data are given as percentages.

*χ2-test (nominal data) or Mann-Whitney test (interval data) were performed

**Intake of oral contraceptives or hormone replacement therapy.

**Fig 1:**
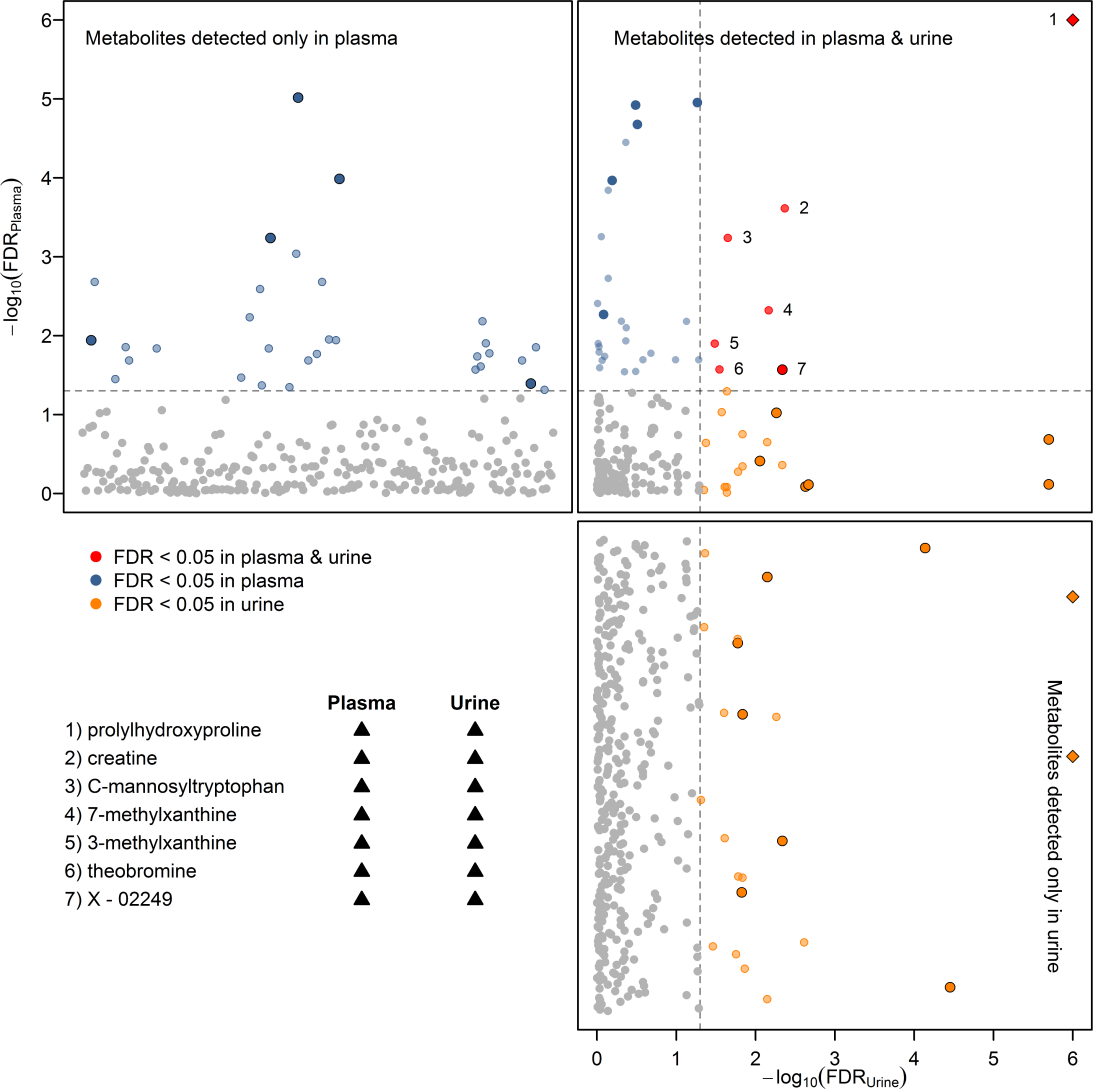
Corrected p-values (false discovery rate; FDR) from linear regression analyses with osteocalcin concentration as exposure and plasma (left upper panel) or urine (right lower panel) metabolites as outcomes. Models were adjusted for age, sex, waist circumference and physical activity. The upper right panel contains results for metabolites present in both fluids. Within each panel the dotted lines denote the significance threshold of FDR<0.05. Metabolites are colored if they are significantly associated in plasma (blue), urine (orange) or in both (red), respectively. Metabolites marked with a diamond exceed the plotting range. Metabolites highlighted by increased point size and darker colors persisted significant even after further adjustment for hormone intake, estimated glomerular filtration rate and smoking behavior. Corresponding beta estimates and FDR-values can be found in supplemental Tables 1 and 2. Metabolites numbered with 1 to 7 are named in the lower left panel. Triangles indicate the direction of the association in plasma or urine, with ▲ indicating positive associations.

We calculated age, sex, WC, and physical activity adjusted linear regression models to assess the associations between serum OCN and plasma or urine metabolites and revealed 64 and 48 significantly related metabolites in plasma and urine, respectively (Fig 1).

These associations included seven metabolites present in both, plasma and urine, e.g. prolylhydroxyproline (PHP) and C-mannosyltryptophan. In particular the latter one pointed towards an important role of the kidneys in these relations, as highlighted by a recent metabolomics study [14]. This was further supported by a weak inverse correlation between serum OCN and the eGFR (r = -0.17; p<0.001). Therefore, we reanalyzed all models after additional adjustment for the eGFR and further covariates (see methods) and observed an important drop in the number of associated metabolites (S1 Fig, S1 and S2 Tables).

**Fig 2:**
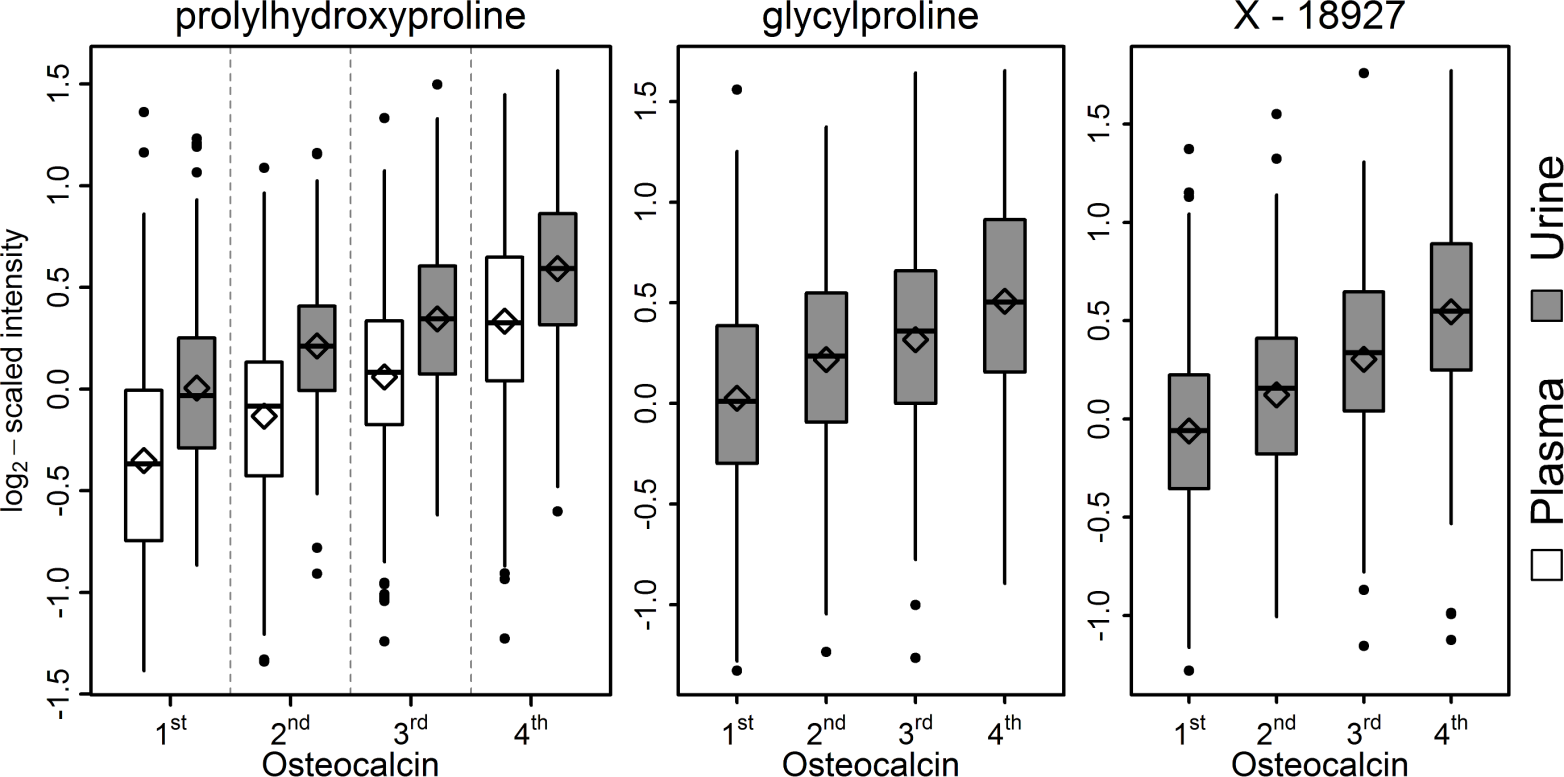
Boxplots for the four most significantly associated metabolites according to quartiles of serum osteocalcin concentration.

After the additional adjustment twelve plasma and 17 urine metabolites remained significantly associated with OCN. Among them, three metabolites clearly stood out, the proline metabolites PHP (plasma and urine) and glycylproline (urine) as well as the unknown metabolite X-18927 (urine) (Fig 2 and Table 2). All three metabolites showed a positive association with serum OCN. Interestingly, X-18927 showed a strong correlation with the two proline derivatives (S2 Fig) and hence might point towards a functional relation.

**Table 2:** Plasma and urine metabolites significantly associated with the serum osteocalcin concentration.

Metabolite	Class	N	β (95%-CI)*	FDR
**Plasma**				
2-aminoheptanoate	Lipid	879	0.164 (0.076; 0.252)	2.55E-02
3-hydroxyisobutyrate	Amino Acid	905	-0.207 (-0.308; -0.106)	1.54E-02
3-methyl-2-oxobutyrate	Amino Acid	910	-0.091 (-0.143; -0.039)	3.02E-02
pipecolate	Amino Acid	900	-0.192 (-0.289; -0.095)	1.81E-02
kynurenine	Amino Acid	908	0.092 (0.041; 0.143)	2.85E-02
citrulline	Amino Acid	909	0.096 (0.044; 0.149)	2.55E-02
prolylhydroxyproline	Amino Acid	909	0.706 (0.629; 0.784)	1.20E-58
phenylalanyltryptophan	Peptide	852	0.147 (0.062; 0.231)	3.02E-02
N-acetylcarnosine	Peptide	701	0.162 (0.078; 0.246)	1.93E-02
X—11378	Unknown	908	0.168 (0.071; 0.265)	3.02E-02
X—12104	Unknown	758	-0.136 (-0.213; -0.059)	3.02E-02
X—16394	Unknown	907	0.097 (0.040; 0.154)	3.51E-02
**Urine**				
tiglyl carnitine	Amino Acid	921	-0.151 (-0.223; -0.078)	4.83E-03
prolylhydroxyproline	Amino Acid	921	0.603 (0.537; 0.668)	2.31E-60
riboflavin (Vitamin B2)	Cof. and Vit.	900	-0.305 (-0.487; -0.122)	4.12E-02
glycylproline	Peptide	912	0.508 (0.424; 0.591)	3.02E-28
HPMA	Xenobiotics	845	-0.284 (-0.451; -0.116)	3.73E-02
hydroxycotinine	Xenobiotics	448	-1.228 (-1.887; -0.569)	1.57E-02
1-methylurate	Xenobiotics	908	0.316 (0.134; 0.498)	2.93E-02
X—02249	Unknown	900	0.373 (0.180; 0.566)	1.15E-02
X—12095	Unknown	918	-0.155 (-0.242; -0.067)	2.41E-02
X—12170	Unknown	917	-0.134 (-0.205; -0.064)	1.15E-02
X—12689	Unknown	919	-0.207 (-0.293; -0.121)	3.81E-04
X—13840	Unknown	609	-0.592 (-0.919; -0.265)	2.10E-02
X—12511	Unknown	921	0.280 (0.144; 0.417)	4.83E-03
X—17306	Unknown	751	-0.343 (-0.550; -0.136)	4.13E-02
X—17308	Unknown	917	0.203 (0.117; 0.290)	4.70E-04
X—18927	Unknown	819	0.596 (0.507; 0.685)	1.55E-33
X—21201	Unknown	666	-0.265 (-0.428; -0.102)	4.85E-02

95%-CI = 95% confidence interval; FDR = false discovery rate to correct for multiple testing; HPMA = S-(3-hydroxypropyl)mercapturic acid; Cof. and Vits. = Cofactors and Vitamins.

*linear regression models adjusted for age, sex, waist circumference, physical activity, estimated glomerular filtration rate, intake of oral contraceptive or hormone replacement therapy and smoking behavior.

Besides the proline derivatives, serum OCN was positively associated with plasma concentrations of 2-aminoheptanoate, kynurenine, citrulline, phenylalanyltryptophan, N-acteylcarnosine, and two metabolites of unknown identity (Table 2). Inverse associations were restricted to plasma concentrations of 3-hydroxyisobutyrate, 3-methyl-2-oxobutyrate, pipecolate and one unknown metabolite (X—12104). With respect to urine, 1-methylurate (1-MU) and four unknown metabolites showed a positive association with serum OCN. Additionally, serum OCN was inversely associated with urine concentrations of tiglyl carnitine, riboflavin (vitamin B2), the xenobiotics S-(3-hydroxypropyl)mercapturic acid (HPMA) and hydroxycotinine as well as five unknown metabolites.

The exclusion of participants with 25-hydroxy vitamin D levels below 10μg/L did not affect the presented results (S3 Fig).

## Discussion

The present study investigated metabolic effects of OCN based on a multi-fluid non-targeted metabolomics approach in an epidemiological setting. Overall, the serum OCN concentration was significantly related with metabolites involved in bone and energy metabolism as well as in detoxification of xenobiotics.

The unexpectedly huge number of associated metabolites in the first analyses indicated some hidden confounding. Indeed, closer inspection of the associated metabolites indicated a potential role of renal function in the associations of the serum OCN concentration with plasma or urine metabolites, e.g. C-mannosyltrypophan [14]. Former investigations already showed that the clearance of OCN, by metabolism or excretion, is depending on renal function as OCN is freely filtered (molecular size of 5-6kDa) [15]. Consequently, the additional adjustment for renal function (eGFR) led to a reduction in the number of significant findings, which might be considered as independent of renal function.

### The interplay between bone, OCN and metabolism

The most pronounced association with serum OCN was found for PHP in plasma and urine. PHP is a collagen-derived peptide and against this background PHP was discussed as potential new, fast-responding marker for alterations in bone status or metabolism [16]. A study among postmenopausal women [16] confirmed highly significant correlations of serum OCN with urinary PHP as well as of both markers with other markers of bone turnover or dual X-ray absorptiometry (DXA). These results are not surprising as during bone turnover both the collagenous and the non-collagenous parts of the organic matrix are resorbed and newly synthesized, releasing degradation intermediates like PHP. The association of the serum OCN concentration with PHP is further supported by the observed positive association between serum OCN and the urinary glycylproline concentration. Glycylproline is a dipeptide and part of the primary structure of collagen. This finding is most likely due to the concurrent release of both OCN and glycylproline during bone remodeling. For X-18927, an unknown metabolite, no further information is currently available. However, the strong correlation of X-18927 with PHP and glycylproline suggests a functional link between these three metabolites.

Other detected metabolites, for example kynurenine, seem to be linked to OCN and bone metabolism in a rather indirect manner. Kynurenine is a degradation product of tryptophan and was positively associated with OCN in the present study. The Hordaland Health Study [17] discovered a relation between tryptophan metabolism and bone mineral density (BMD) whereby kynurenine showed a positive association to BMD in middle-aged women and men and older women. However, an *in vitro* study revealed opposite findings by reporting that tryptophan increased OCN expression in bone mesenchymal stem cells (BMMSC) and mediates increased proliferation of BMMSC, while kynurenine had no influence on OCN expression and exerts inhibitory effects on BMMSC proliferation [18]. The associations between tryptophan metabolism and BMD or OCN may further be modulated by inflammation. The key step of tryptophan degradation is catalyzed by an enzyme called indoleamin 2,3-dioxygenase 1 (IDO1). The enzyme activity seemed to be increased by inflammation, especially by IFN-γ [17]. In *in vitro* experiments the blockage of *IDO* led to decreased osteoblastogenesis and IDO1 knockout (IDO^-^/^-^) mice showed osteopenia with decreased amounts of osteoblasts and increased amounts of osteoclasts [19]. Possibly, even subclinical inflammation could be able to increase kynurenine concentrations by changing expression of IDO1 and reduce BMD by inducing bone resorption. Increased bone resorption, in turn, could be a potential promoter of increased serum OCN.

Citrulline, which was positively associated with OCN, is a non-proteinogenic amino acid that is either ingested or produced endogenously. It is a side product during arginine degradation in the synthesis of nitric oxide (NO). NO is an important signaling molecule in almost every tissue, including bone [20] and *in vitro* studies indicated a reliance of NO availability for proliferation of osteoblast-like cells [21]. NO also improves blood circulation [22], which may enhance bone nutrition and oxygen supply. Additionally, several previous studies demonstrated a correlation between bone health and cardiovascular disease [23]. Up to now, the mechanisms behind this interplay are not fully understood. The relation between OCN and NO might provide a possible explanation and the basis for future research. Another possible explanation for the association between plasma citrulline and OCN might be found in energy metabolism. As mentioned above, OCN seems to have protective effects towards development of diabetes and the metabolic syndrome. Citrulline treatment *in vitro*, using hepatic cell lines [24], or *in vivo*, in rodent models fed a high-fat diet, improved insulin sensitivity and decreased circulating insulin concentrations [25]. Citrulline also seems to be a predictor for the metabolic syndrome [26] and a mice model simulating type 1 diabetes mellitus showed increased plasma citrulline concentrations [27]. Altogether the anti-diabetic effects of OCN and citrulline seem congruent with each other. However, whether the involved mechanisms are interlinking with each other is speculative. Previous studies revealed that OCN is influencing K^+^_V_-channels [28] and GPRC6A [29] in pancreatic islet cells, but the exact role of citrulline in energy metabolism remains unclear.

### OCN and surrogates of lifestyle

In the present study, we observed a negative relation between OCN and urinary riboflavin, known as vitamin B2, a metabolite that cannot be synthesized endogenously. A previous study investigating more than 5300 elderly subjects proposed that a higher dietary riboflavin intake leads to slightly higher BMD in the femoral neck and lumbar spine but not to a reduced fracture risk [30] which is in contrast to our findings. However, the inverse associations observed here might be due to individual capacities of vitamin B2 uptake from the intestine or successful reabsorption within the kidneys which would finally lead to a decreased vitamin B2 excretion. Unfortunately, plasma concentrations of vitamin B2 were not assessed in the present study and hence we can only speculate about the endogenous availability of vitamin B2.

OCN was also inversely associated with urinary hydroxycotinine, a metabolite derived from tobacco metabolism [31]. Moreover, OCN was also linked to S-(3-hydroxypropyl)mercapturic acid (HPMA) another metabolite related to smoking. HPMA, as intermediate in acrolein metabolism, is a toxic and carcinogenic agent common in tobacco, food and the environment [32]. A Turkish study discovered decreased serum OCN concentrations in pregnant smoking women as well as in umbilical blood of their newborns. Interestingly, the effect on newborns is even demonstrable among mothers passively exposed to smoke [33] but an explanation is still outstanding. With respect to bone health, previous studies showed that smoking is a risk factor for osteoporosis [34] and is related to impaired BMD in older men [35] and premenopausal women [36]. Several mechanisms were proposed [34] which might explain the association between smoking and impaired BMD. Cigarette smoking impairs collagen synthesis and alters the circulating amount of several growth factors. Moreover, smoking influences the production and secretion of sexual, calciotropic and adrenocortical hormones. Altered concentrations of these hormones are also known to be related to an impaired BMD.

Beside the xenobiotic hydroxycotinine, the urinary concentration of 1-MU was positively related with OCN. 1-MU is a metabolite of caffeine or theophylline and therefore detectable in coffee or tea drinkers [37]. Coffee consumption seems to have multiple effects on bone metabolism. Long-term coffee consumption was associated with a slightly impaired BMD among Swedish women, but not with an increased fracture rate [38]. An *in vivo* investigation in rodents showed that caffeine also seems to increase osteoclast numbers [39]. These data are consistent with the present result, considering that higher circulating OCN concentrations may be related to a reduced BMD in elderly subjects [40, 41].

### OCN associations distinct from bone metabolism

The inverse relation between OCN and pipecolate cannot be explained directly, but *via* its source lysine [42]. Lysine seems to boost osteoblast proliferation but an immediate influence of lysine on OCN could not be detected [43]. Apart from that, lysine is an activator of GPRC6A, the OCN receptor [44]. Decreased plasma concentrations of pipecolate might thus indicate diminished lysine degradation which in turn might provoke an attenuated induction of GPRC6A and hence higher OCN concentrations. However, plasma lysine concentration was not related to the serum OCN concentration in the present study and further studies are required to elucidate a possible mechanism behind this association.

Besides pipecolate, serum OCN was inversely related to intermediates of valine degradation, namely 3-hydroxyisobutyrate (3HIB) and 3-methyl-2-oxobutyrate (3M2O), pointing towards an implication on energy metabolism and glucose turnover. 3HIB was shown to promote trans-endothelial fatty acid (FA) transport and subsequent accumulation of fat in muscle tissue favoring local insulin resistance [45]. OCN deficient mice demonstrated decreased glucose tolerance and accumulation of body fat [3], which can also be provoked by insulin resistance. Nevertheless, OCN seems to have rather global effects on energy metabolism by influencing pancreatic beta cells via the GPRC6A [29] receptor and inhibiting K^+^_V_-channels [28]. These channels are inhibited by ATP in times of sufficient glucose supply and therefore essential for the regulation of insulin release. Further research is necessary to investigate whether the systemic effects of OCN and the local effects of 3HIB are functionally related. 3M2O is located further upstream in the valine degradation pathway than 3HIB. This strengthens the indication towards a possible functional relation between serum OCN and valine degradation.

### Strengths and limitations

Our study has strengths and some potential limitations. Metabolomics is a powerful tool for clinical research, since it is able to compress influences from genetics, environment, health behavior and interventions in intermediate phenotypes, which consist of final downstream products of various pathways. Since our findings depend on study region and measurement procedures they should be regarded as hypothesis generating. Replication in other cohorts and pharmacological intervention studies are needed to confirm our results and to obtain possible causal relations. Furthermore, epidemiological studies, especially cross-sectional analyses, are generally not suitable to prove causal relations.

## Conclusion

We firstly investigated the interrelations between serum OCN and the plasma and urine metabolome by untargeted MS data in a large study. The discovered metabolites were, as expected, mostly related to bone and energy metabolism with several metabolites assignable to both fields. However, most of the metabolites were previously unknown to be related to OCN. Altogether our results highlight the role of OCN in bone metabolism and support, although in a limited way, an influence of OCN on whole-body metabolism. Further experimental work is necessary to investigate the interaction between OCN and the discovered metabolites, especially those related to energy homeostasis.

## Supporting information

S1 Fig Comparison of adjusted p-values (false discovery rate; FDR) from linear regression analyses with osteocalcin concentration as exposure and plasma (left panel) or urine metabolites as outcome using two different models. The first model (model1) was adjusted for age, sex, waist circumference and physical activity. The second model was additionally adjusted for the intake of oral contraceptives or hormone replacement therapy as well as the estimated glomerular filtration rate. Metabolites colored in red were significantly associated in both models whereas yellow ones were signficantly associated only in one of them. Corresponding beta estimates and FDR-values can be found in Tables 2, S1 and S2.
(PDF)

S2 Fig Matrix of scatterplots comparing correlations between plasma and urine concentration of prolylhydroxyproline (PHP), urine concentration of glycylproline as well as the unknown metabolite X– 18927. The upper triangle of the matrix includes the simple scatterplots, whereas the lower triangle indicates corresponding Pearson correlation coefficients. The latter were all highly significant (p<0.0001).
(PDF)

S3 Fig Comparison of adjusted p-values (false discovery rate; FDR) from linear regression analyses with osteocalcin concentration as exposure and plasma (left panel) or urine metabolites as outcome using two different populations. The x-axis displays results from the whole study population (all) whereas the y-axis contains results from a subpopulation after excluding participants with low 25-hydroxy vitamin D levels (<10 μg/l). Metabolites colored in red were significantly associated in both populations whereas yellow ones only in one of them.\
(PDF)

S1 Table Comparison of significantly associated plasma metabolites using different linear models. (PDF)

S2 Table Comparison of significantly associated urine metabolites using different linear models. (PDF)

S1 Appendix (PDF)
