# Supplementary material for: Comprehensive metabolic characterization of serum osteocalcin action in a large non-diabetic sample
Source: PLoS One. 2017 Sep 18;12(9):e0184721. doi: 10.1371/journal.pone.0184721 (PMC5602537; doi:10.1371/journal.pone.0184721)
Supplement: S1 Fig — The first model (model1) was adjusted for age, sex, waist circumference and physical activity. The second model was additionally adjusted for the intake of oral contraceptives or hormone replacement therapy as well as the estimated glomerular filtration rate. Metabolites colored in red were significantly associated in both models whereas yellow ones were signficantly associated only in one of them. Corresponding beta estimates and FDR-values can be found in Tables 2, S1 and S2. (PDF) [file pone.0184721.s001.pdf]

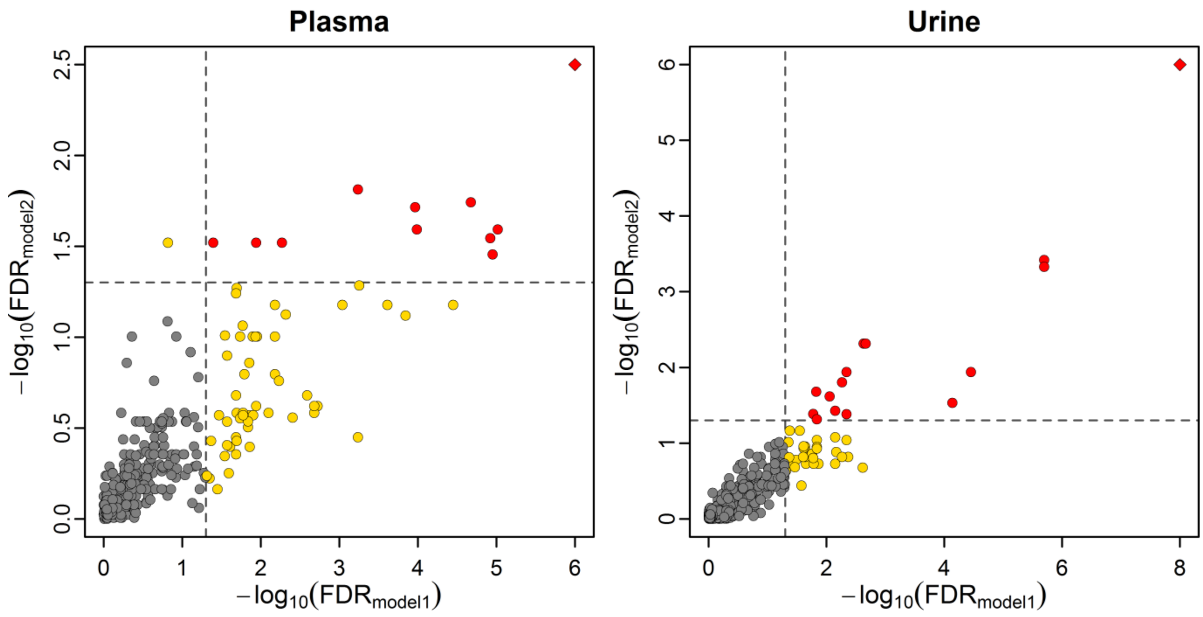

S1 Figure. Comparison of adjusted p-values (false discovery rate; FDR) from linear regression analyses with osteocalcin concentration as exposure and plasma (left panel) or urine metabolites as outcome using two different models. The first model (model1) was adjusted for age, sex, waist circumference and physical activity. The second model was additionally adjusted for the intake of oral contraceptive or hormone replacement therapy as well as the estimated glomerular filtration rate. Metabolites colored in red were significantly associated in both models whereas yellow ones only in one of them. Corresponding beta estimates and FDR-values can be found in table 2, S1 and S2.
