# Supplementary material for: Comprehensive metabolic characterization of serum osteocalcin action in a large non-diabetic sample
Source: PLoS One. 2017 Sep 18;12(9):e0184721. doi: 10.1371/journal.pone.0184721 (PMC5602537; doi:10.1371/journal.pone.0184721)
Supplement: S2 Fig — The upper triangle of the matrix includes the simple scatterplots, whereas the lower triangle indicates corresponding Pearson correlation coefficients. The latter were all highly significant (p<0.0001). (PDF) [file pone.0184721.s002.pdf]

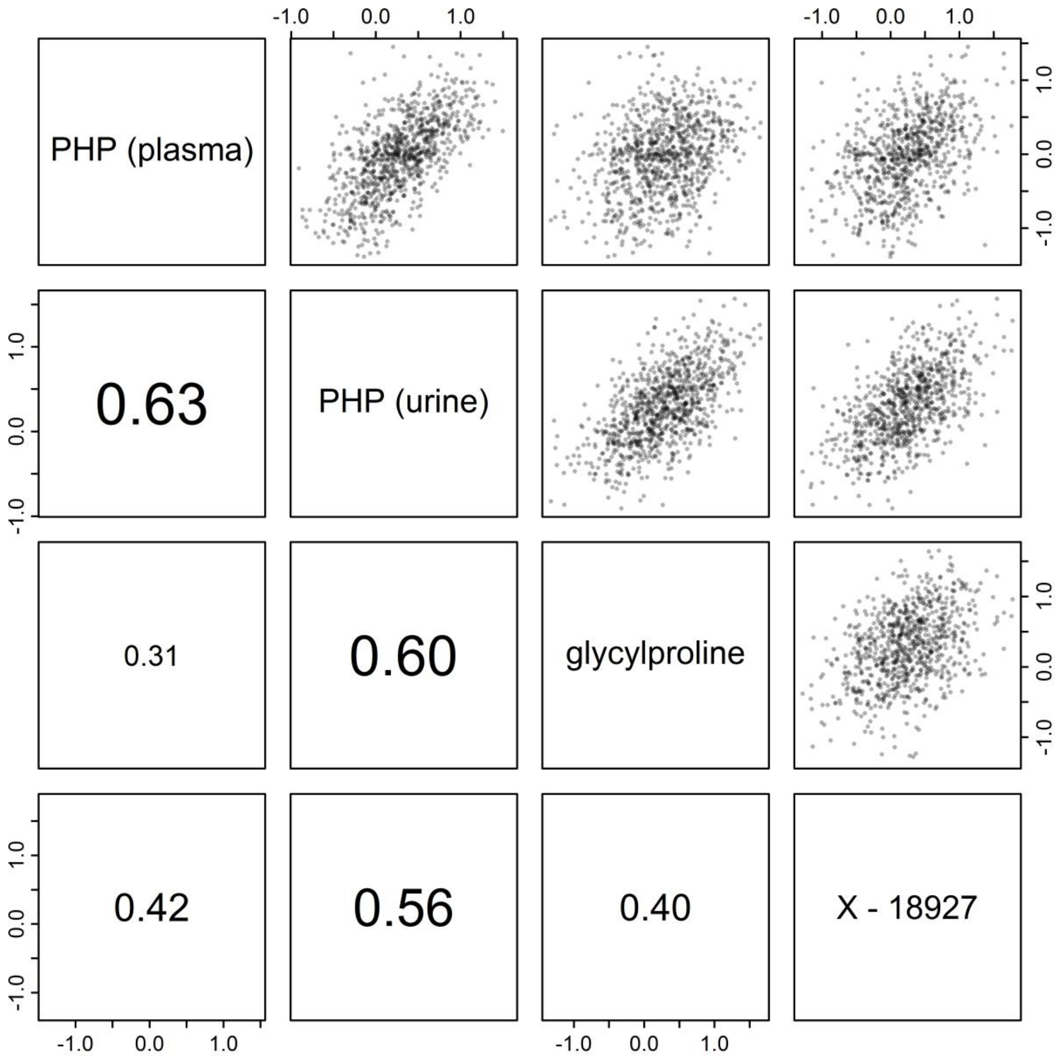

S2 Figure. Figure S2. Matrix of scatterplots comparing correlations between plasma and urine concentrations of prolylhydroxyproline (PHP), urine concentration of glycylproline as well as the unknown metabolite X – 18927. The upper triangle of the matrix includes the simple scatterplots, whereas the lower triangle indicates corresponding Pearson correlation coefficients. The latter were all highly significant ($p<0.0001$).
