# Supplementary material for: Comprehensive metabolic characterization of serum osteocalcin action in a large non-diabetic sample
Source: PLoS One. 2017 Sep 18;12(9):e0184721. doi: 10.1371/journal.pone.0184721 (PMC5602537; doi:10.1371/journal.pone.0184721)
Supplement: S3 Fig — The x-axis displays results from the whole study population (all) whereas the y-axis contains results from a subpopulation after excluding participants with low 25-hydroxy vitamin D levels (<10 μg/l). Metabolites colored in red were significantly associated in both populations whereas yellow ones only in one of them.\ (PDF) [file pone.0184721.s003.pdf]

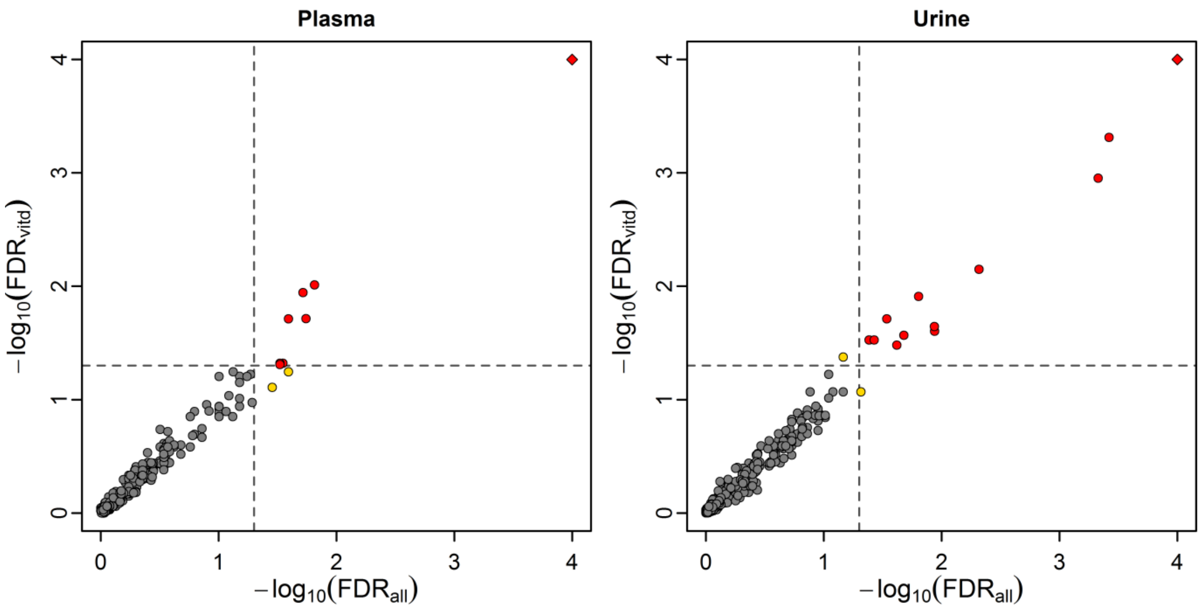

S3 Figure. Comparison of adjusted p-values (false discovery rate; FDR) from linear regression analyses with osteocalcin concentration as exposure and plasma (left panel) or urine metabolites as outcome using two different populations. The x-axis displays results from the whole study population (all) whereas the y-axis contains results from a subpopulation after excluding participants with low 25-hydroxy vitamin D levels (<10 µg/l). Metabolites colored in red were significantly associated in both populations whereas yellow ones only in one of them.
