# Supplementary material for: Comprehensive metabolic characterization of serum osteocalcin action in a large non-diabetic sample
Source: PLoS One. 2017 Sep 18;12(9):e0184721. doi: 10.1371/journal.pone.0184721 (PMC5602537; doi:10.1371/journal.pone.0184721)
Supplement: S1 Table — (PDF) [file pone.0184721.s004.pdf]

Supplemental Table 1 Comparison of signficantly associted plasma metabolites using different linear models.

Metabolite1	Class	Pathway Assignment	N	model 1 β (95%-CI)	model 1 FDR	model2 β (95%-CI)	model2 FDR
N-acetylalanine	Amino Acid	Alanine and Aspartate Metabolism	903	0.080 (0.040; 0.120)	2.57E-03	0.048 (0.008; 0.089)	2.09E-01
creatine	Amino Acid	Creatine Metabolism	911	-0.235 (-0.335; -0.134)	2.44E-04	-0.159 (-0.263; -0.056)	6.64E-02
creatinine	Amino Acid	Creatine Metabolism	910	0.056 (0.016; 0.096)	4.28E-02	0.036 (-0.003; 0.074)	3.73E-01
glutamine	Amino Acid	Glutamate Metabolism	907	0.071 (0.039; 0.103)	5.57E-04	0.054 (0.021; 0.087)	5.20E-02
5-oxoproline	Amino Acid	Glutathione Metabolism	910	0.054 (0.020; 0.088)	2.03E-02	0.040 (0.004; 0.075)	2.61E-01
betaine	Amino Acid	Glycine, Serine and Threonine Metabolism	905	0.095 (0.038; 0.152)	1.45E-02	0.057 (-0.000; 0.115)	3.13E-01
**3-methyl-2-oxobutyrate**	Amino Acid	Leucine, Isoleucine and Valine Metabolism	910	-0.093 (-0.143; -0.044)	5.39E-03	-0.091 (-0.143; -0.039)	3.02E-02
4-methyl-2-oxopentanoate	Amino Acid	Leucine, Isoleucine and Valine Metabolism	910	-0.081 (-0.132; -0.031)	1.84E-02	-0.073 (-0.126; -0.021)	9.92E-02
**pipecolate**	Amino Acid	Lysine Metabolism	900	-0.253 (-0.348; -0.158)	2.11E-05	-0.192 (-0.289; -0.095)	1.81E-02
3-methoxytyrosine	Amino Acid	Phenylalanine and Tyrosine Metabolism	869	0.121 (0.055; 0.187)	6.59E-03	0.086 (0.018; 0.153)	1.60E-01
3-(4-hydroxyphenyl)lactate	Amino Acid	Phenylalanine and Tyrosine Metabolism	863	0.098 (0.028; 0.168)	4.49E-02	0.044 (-0.027; 0.115)	5.99E-01
acisoga	Amino Acid	Polyamine Metabolism	911	0.116 (0.043; 0.189)	2.03E-02	0.070 (-0.006; 0.145)	3.73E-01
**kynurenine**	Amino Acid	Tryptophan Metabolism	908	0.140 (0.089; 0.191)	1.20E-05	0.092 (0.041; 0.143)	2.85E-02
C-glycosyltryptophan*	Amino Acid	Tryptophan Metabolism	908	0.095 (0.052; 0.138)	5.78E-04	0.037 (-0.002; 0.076)	3.56E-01
proline	Amino Acid	Urea cycle; Arginine and Proline Metabolism	908	0.125 (0.067; 0.183)	9.17E-04	0.094 (0.033; 0.154)	6.64E-02
**citrulline**	Amino Acid	Urea cycle; Arginine and Proline Metabolism	909	0.145 (0.094; 0.197)	9.60E-06	0.096 (0.044; 0.149)	2.55E-02
**pro-hydroxy-pro**	Amino Acid	Urea cycle; Arginine and Proline Metabolism	909	0.757 (0.681; 0.833)	4.56E-69	0.706 (0.629; 0.784)	1.20E-58
1,5-anhydroglucitol (1,5-AG)	Carbohydrate	Glycolysis, Gluconeogenesis, and Pyruvate Metabolism	904	0.102 (0.034; 0.170)	2.85E-02	0.103 (0.031; 0.174)	9.80E-02
pyruvate	Carbohydrate	Glycolysis, Gluconeogenesis, and Pyruvate Metabolism	906	-0.141 (-0.232; -0.051)	2.06E-02	-0.150 (-0.245; -0.056)	5.75E-02
nicotinamide	Cofactors and Vitamins	Nicotinate and Nicotinamide Metabolism	862	-0.171 (-0.277; -0.066)	1.70E-02	-0.161 (-0.271; -0.051)	8.65E-02
citrate	Energy	TCA Cycle	910	0.058 (0.026; 0.090)	6.61E-03	0.047 (0.014; 0.080)	9.92E-02
deoxycarnitine	Lipid	Carnitine Metabolism	893	0.101 (0.051; 0.150)	2.09E-03	0.059 (0.008; 0.110)	2.39E-01
octanoylcarnitine	Lipid	Fatty Acid Metabolism(Acyl Carnitine)	903	0.170 (0.070; 0.269)	1.26E-02	0.146 (0.043; 0.249)	9.92E-02
decanoylcarnitine	Lipid	Fatty Acid Metabolism(Acyl Carnitine)	905	0.181 (0.077; 0.284)	1.12E-02	0.148 (0.041; 0.256)	9.92E-02
cis-4-decenoyl carnitine	Lipid	Fatty Acid Metabolism(Acyl Carnitine)	898	0.171 (0.072; 0.270)	1.14E-02	0.118 (0.016; 0.221)	2.39E-01
2-aminooctanoate	Lipid	Fatty Acid, Amino	900	0.169 (0.065; 0.273)	1.68E-02	0.119 (0.011; 0.227)	2.69E-01
**2-aminoheptanoate**	Lipid	Fatty Acid, Amino	879	0.211 (0.125; 0.296)	1.03E-04	0.164 (0.076; 0.252)	2.55E-02
arachidonate (20:4n6)	Lipid	Polyunsaturated Fatty Acid (n3 and n6)	911	-0.079 (-0.131; -0.026)	2.69E-02	-0.055 (-0.109; -0.001)	2.92E-01
dihomo-linoleate (20:2n6)	Lipid	Polyunsaturated Fatty Acid (n3 and n6)	907	-0.144 (-0.234; -0.054)	1.84E-02	-0.099 (-0.192; -0.007)	2.79E-01
docosahexaenoate (DHA; 22:6n3)	Lipid	Polyunsaturated Fatty Acid (n3 and n6)	906	-0.136 (-0.224; -0.047)	2.46E-02	-0.080 (-0.170; 0.010)	4.00E-01
docosadienoate (22:2n6)	Lipid	Polyunsaturated Fatty Acid (n3 and n6)	896	-0.173 (-0.268; -0.079)	6.59E-03	-0.152 (-0.250; -0.054)	6.64E-02
adrenate (22:4n6)	Lipid	Polyunsaturated Fatty Acid (n3 and n6)	908	-0.124 (-0.197; -0.052)	1.25E-02	-0.082 (-0.157; -0.007)	2.69E-01
dihomo-linolenate (20:3n3 or n6)	Lipid	Polyunsaturated Fatty Acid (n3 and n6)	909	-0.096 (-0.155; -0.037)	1.68E-02	-0.068 (-0.128; -0.007)	2.61E-01
cortisol	Lipid	Steroid	909	-0.188 (-0.280; -0.097)	1.89E-03	-0.105 (-0.196; -0.015)	2.39E-01
cortisone	Lipid	Steroid	905	-0.085 (-0.135; -0.034)	1.38E-02	-0.046 (-0.098; 0.006)	4.02E-01
4-androsten-3beta,17beta-diol disulfate (1)*	Lipid	Steroid	905	-0.283 (-0.447; -0.119)	1.17E-02	-0.234 (-0.404; -0.065)	9.92E-02
N6-carbamoylthreonyladenosine	Nucleotide	Purine Metabolism, Adenine containing	853	0.108 (0.052; 0.163)	3.92E-03	0.060 (0.004; 0.116)	2.77E-01
pseudouridine	Nucleotide	Pyrimidine Metabolism, Uracil containing	904	0.098 (0.060; 0.135)	3.56E-05	0.055 (0.019; 0.091)	6.64E-02
5-methyluridine (ribothymidine)	Nucleotide	Pyrimidine Metabolism, Uracil containing	698	0.078 (0.028; 0.128)	2.06E-02	0.050 (-0.003; 0.103)	3.56E-01
**phenylalanyltryptophan**	Peptide	Dipeptide	852	0.117 (0.035; 0.199)	4.04E-02	0.147 (0.062; 0.231)	3.02E-02
**N-acetylcarnosine**	Peptide	Dipeptide Derivative	701	0.200 (0.118; 0.281)	1.08E-04	0.162 (0.078; 0.246)	1.93E-02
gamma-glutamyltyrosine	Peptide	Gamma-glutamyl Amino Acid	908	0.140 (0.082; 0.198)	1.44E-04	0.089 (0.030; 0.148)	7.60E-02
gamma-glutamylphenylalanine	Peptide	Gamma-glutamyl Amino Acid	907	0.066 (0.023; 0.110)	2.56E-02	0.030 (-0.014; 0.074)	5.61E-01
gamma-glutamylisoleucine*	Peptide	Gamma-glutamyl Amino Acid	889	0.099 (0.033; 0.165)	2.88E-02	0.055 (-0.013; 0.122)	4.51E-01
bradykinin	Peptide	Polypeptide	745	-0.490 (-0.782; -0.197)	1.40E-02	-0.330 (-0.633; -0.028)	2.69E-01
bradykinin, des-arg(9)	Peptide	Polypeptide	511	-0.579 (-0.996; -0.162)	4.88E-02	-0.278 (-0.703; 0.148)	5.79E-01
O-sulfo-L-tyrosine	Xenobiotics	Chemical	904	0.092 (0.033; 0.151)	2.06E-02	0.051 (-0.010; 0.111)	4.42E-01
theobromine	Xenobiotics	Xanthine Metabolism	907	0.279 (0.095; 0.464)	2.68E-02	0.256 (0.064; 0.448)	1.26E-01
3-methylxanthine	Xenobiotics	Xanthine Metabolism	687	0.241 (0.099; 0.382)	1.26E-02	0.207 (0.060; 0.353)	9.92E-02
7-methylxanthine	Xenobiotics	Xanthine Metabolism	800	0.279 (0.133; 0.426)	4.79E-03	0.229 (0.077; 0.382)	7.51E-02
X - 02249	Unknown	Unknown	833	0.171 (0.058; 0.285)	2.69E-02	0.106 (-0.011; 0.223)	3.93E-01
**X - 11378**	Unknown	Unknown	908	0.161 (0.068; 0.253)	1.14E-02	0.168 (0.071; 0.265)	3.02E-02
X - 11429	Unknown	Unknown	901	0.102 (0.052; 0.152)	2.09E-03	0.055 (0.006; 0.104)	2.61E-01
X - 11564	Unknown	Unknown	729	0.075 (0.023; 0.126)	3.55E-02	0.026 (-0.026; 0.078)	6.88E-01
X - 11795	Unknown	Unknown	908	0.128 (0.052; 0.204)	1.40E-02	0.104 (0.025; 0.184)	1.39E-01
X - 11880	Unknown	Unknown	910	0.145 (0.053; 0.237)	2.06E-02	0.115 (0.019; 0.211)	2.09E-01
X - 12100	Unknown	Unknown	904	0.105 (0.047; 0.163)	7.97E-03	0.066 (0.007; 0.126)	2.61E-01
**X - 12104**	Unknown	Unknown	758	-0.082 (-0.158; -0.007)	1.52E-01	-0.136 (-0.213; -0.059)	3.02E-02
X - 13435	Unknown	Unknown	910	0.136 (0.054; 0.218)	1.45E-02	0.087 (0.004; 0.171)	2.91E-01
X - 13837	Unknown	Unknown	398	0.249 (0.092; 0.407)	2.03E-02	0.269 (0.103; 0.436)	5.37E-02
**X - 16394**	Unknown	Unknown	907	0.162 (0.103; 0.220)	1.11E-05	0.097 (0.040; 0.154)	3.51E-02
X - 16397	Unknown	Unknown	725	0.135 (0.053; 0.218)	1.62E-02	0.109 (0.023; 0.194)	1.60E-01
X - 19141	Unknown	Unknown	806	0.142 (0.045; 0.239)	3.40E-02	0.112 (0.010; 0.213)	2.69E-01
X - 20845	Unknown	Unknown	392	-0.846 (-1.298; -0.393)	5.85E-03	-0.579 (-1.044; -0.113)	1.74E-01

model1 = lineare regression model adjusted for age, sex, waist circumference and physical activity; model2 = model1 + estimated glomerular filtration rate, the intake of oral contraceptive or hormone replacement therapy and smoking behavior;*Metabolites were annotated based on fragmentation spectra; FDR = false discovery rate; metabolites printed in bold were significantly associated in model2
