# Supplementary material for: Comprehensive metabolic characterization of serum osteocalcin action in a large non-diabetic sample
Source: PLoS One. 2017 Sep 18;12(9):e0184721. doi: 10.1371/journal.pone.0184721 (PMC5602537; doi:10.1371/journal.pone.0184721)
Supplement: S2 Table — (PDF) [file pone.0184721.s005.pdf]

Supplemental Table 2 Comparison of signficantly associted urine metabolites using different linear models.

				model 1		model2	
**Metabolite1**	**Class**	**Pathway Assignment**	**N**	**β (95%-CI)**	**FDR**	**β (95%-CI)**	**FDR**
creatine	Amino Acid	Creatine Metabolism	912	-0.388 (-0.580; -0.195)	4.28E-03	-0.255 (-0.454; -0.056)	1.53E-01
**tiglyl carnitine**	Amino Acid	Leucine, Isoleucine and Valine Metabolism	921	-0.149 (-0.219; -0.079)	2.16E-03	-0.151 (-0.223; -0.078)	4.83E-03
C-glycosyltryptophan*	Amino Acid	Tryptophan Metabolism	920	0.055 (0.021; 0.088)	2.24E-02	0.040 (0.006; 0.074)	1.87E-01
homocitrulline	Amino Acid	Urea cycle; Arginine and Proline Metabolism	917	-0.140 (-0.222; -0.058)	1.66E-02	-0.099 (-0.182; -0.015)	1.87E-01
**pro-hydroxy-pro**	Amino Acid	Urea cycle; Arginine and Proline Metabolism	921	0.606 (0.543; 0.669)	3.55E-65	0.603 (0.537; 0.668)	2.31E-60
lactate	Carbohydrate	Glycolysis, Gluconeogenesis, and Pyruvate Metabolism	913	-0.223 (-0.363; -0.082)	2.67E-02	-0.135 (-0.280; 0.011)	3.64E-01
N1-Methyl-2-pyridone-5-carboxamide	Cofactors and Vitamins	Nicotinate and Nicotinamide Metabolism	919	-0.134 (-0.224; -0.045)	4.22E-02	-0.145 (-0.238; -0.052)	6.84E-02
**riboflavin (Vitamin B2)**	Cofactors and Vitamins	Riboflavin Metabolism	900	-0.298 (-0.474; -0.122)	1.68E-02	-0.305 (-0.487; -0.122)	4.12E-02
gamma-CEHC glucuronide*	Cofactors and Vitamins	Tocopherol Metabolism	921	0.199 (0.081; 0.317)	1.68E-02	0.154 (0.032; 0.277)	1.57E-01
pyridoxate	Cofactors and Vitamins	Vitamin B6 Metabolism	910	-0.142 (-0.229; -0.054)	2.32E-02	-0.130 (-0.222; -0.039)	1.10E-01
sebacate (decanedioate)	Lipid	Fatty Acid, Dicarboxylate	680	0.230 (0.075; 0.385)	4.44E-02	0.241 (0.076; 0.406)	9.71E-02
androsterone sulfate	Lipid	Steroid	917	0.308 (0.132; 0.485)	1.46E-02	0.272 (0.089; 0.455)	9.09E-02
dehydroisoandrosterone sulfate (DHEA-S)	Lipid	Steroid	896	0.539 (0.209; 0.869)	2.29E-02	0.470 (0.127; 0.814)	1.24E-01
andro steroid monosulfate 2*	Lipid	Steroid	908	0.314 (0.102; 0.526)	4.46E-02	0.262 (0.041; 0.483)	1.87E-01
**glycylproline**	Peptide	Dipeptide	912	0.467 (0.386; 0.548)	8.86E-26	0.508 (0.424; 0.591)	3.02E-28
**S-(3-hydroxypropyl)mercapturic acid (HPMA)**	Xenobiotics	Chemical	845	-0.317 (-0.486; -0.147)	7.11E-03	-0.284 (-0.451; -0.116)	3.73E-02
**hydroxycotinine**	Xenobiotics	Tobacco Metabolite	448	-1.326 (-2.007; -0.646)	5.45E-03	-1.228 (-1.887; -0.569)	1.57E-02
cotinine N-oxide	Xenobiotics	Tobacco Metabolite	462	-0.555 (-0.927; -0.184)	4.33E-02	-0.459 (-0.822; -0.096)	1.54E-01
theobromine	Xenobiotics	Xanthine Metabolism	918	0.305 (0.111; 0.500)	2.85E-02	0.317 (0.114; 0.520)	6.84E-02
3-methylxanthine	Xenobiotics	Xanthine Metabolism	914	0.232 (0.082; 0.382)	3.27E-02	0.193 (0.037; 0.349)	1.67E-01
7-methylxanthine	Xenobiotics	Xanthine Metabolism	913	0.270 (0.128; 0.412)	6.83E-03	0.201 (0.053; 0.349)	1.31E-01
**1-methylurate**	Xenobiotics	Xanthine Metabolism	908	0.444 (0.268; 0.620)	7.27E-05	0.316 (0.134; 0.498)	2.93E-02
**X - 02249**	Unknown	Unknown	900	0.366 (0.181; 0.551)	4.59E-03	0.373 (0.180; 0.566)	1.15E-02
X - 01911	Unknown	Unknown	914	-0.353 (-0.541; -0.165)	7.11E-03	-0.298 (-0.494; -0.101)	8.36E-02
X - 10593	Unknown	Unknown	920	0.175 (0.074; 0.276)	1.46E-02	0.148 (0.043; 0.253)	1.12E-01
X - 12026	Unknown	Unknown	913	0.184 (0.086; 0.282)	7.11E-03	0.121 (0.020; 0.223)	1.87E-01
**X - 12095**	Unknown	Unknown	918	-0.153 (-0.237; -0.070)	8.81E-03	-0.155 (-0.242; -0.067)	2.41E-02
X - 12096	Unknown	Unknown	920	0.231 (0.089; 0.372)	2.29E-02	0.189 (0.041; 0.337)	1.53E-01
**X - 12170**	Unknown	Unknown	917	-0.177 (-0.245; -0.109)	3.53E-05	-0.134 (-0.205; -0.064)	1.15E-02
X - 12339	Unknown	Unknown	912	-0.188 (-0.294; -0.082)	1.37E-02	-0.132 (-0.242; -0.022)	1.87E-01
**X - 12689**	Unknown	Unknown	919	-0.240 (-0.322; -0.157)	2.01E-06	-0.207 (-0.293; -0.121)	3.81E-04
X - 12687	Unknown	Unknown	915	0.215 (0.087; 0.343)	1.76E-02	0.178 (0.044; 0.311)	1.38E-01
X - 12739	Unknown	Unknown	918	0.174 (0.061; 0.287)	3.46E-02	0.134 (0.017; 0.252)	2.07E-01
X - 12748	Unknown	Unknown	899	-0.152 (-0.230; -0.075)	4.59E-03	-0.120 (-0.200; -0.040)	9.09E-02
X - 12760	Unknown	Unknown	921	0.226 (0.118; 0.334)	2.44E-03	0.126 (0.015; 0.237)	2.10E-01
**X - 12511**	Unknown	Unknown	921	0.277 (0.146; 0.409)	2.35E-03	0.280 (0.144; 0.417)	4.83E-03
X - 13462	Unknown	Unknown	914	0.076 (0.029; 0.123)	2.45E-02	0.068 (0.019; 0.118)	1.17E-01
**X - 13840**	Unknown	Unknown	609	-0.557 (-0.879; -0.235)	1.49E-02	-0.592 (-0.919; -0.265)	2.10E-02
X - 14445	Unknown	Unknown	906	0.109 (0.047; 0.171)	1.46E-02	0.089 (0.025; 0.154)	1.18E-01
X - 14626	Unknown	Unknown	450	-0.364 (-0.576; -0.151)	1.65E-02	-0.262 (-0.484; -0.039)	1.87E-01
**X - 17306**	Unknown	Unknown	751	-0.399 (-0.600; -0.198)	4.59E-03	-0.343 (-0.550; -0.136)	4.13E-02
**X - 17308**	Unknown	Unknown	917	0.241 (0.158; 0.324)	2.01E-06	0.203 (0.117; 0.290)	4.70E-04
X - 17320	Unknown	Unknown	771	-0.472 (-0.766; -0.178)	2.45E-02	-0.425 (-0.722; -0.128)	1.10E-01
X - 17688	Unknown	Unknown	915	-0.216 (-0.364; -0.068)	4.88E-02	-0.167 (-0.321; -0.013)	2.34E-01
**X - 18927**	Unknown	Unknown	819	0.615 (0.530; 0.700)	9.06E-39	0.596 (0.507; 0.685)	1.55E-33
X - 20643	Unknown	Unknown	916	-0.179 (-0.271; -0.086)	5.46E-03	-0.123 (-0.219; -0.028)	1.52E-01
**X - 21201**	Unknown	Unknown	666	-0.272 (-0.427; -0.116)	1.46E-02	-0.265 (-0.428; -0.102)	4.85E-02
X - 21909	Unknown	Unknown	476	-0.355 (-0.576; -0.133)	2.50E-02	-0.303 (-0.541; -0.065)	1.53E-01

model1 = lineare regression model adjusted for age, sex, waist circumference and physical activity; model2 = model1 + estimated glomerular filtration rate, the intake of oral contraceptive or hormone replacement therapy and smoking behavior;*Metabolites were annotated based on fragmentation spectra; FDR = false discovery rate; metabolites printed in bold were significantly associated in model2
