# Supplementary material for: Comprehensive metabolic characterization of serum osteocalcin action in a large non-diabetic sample
Source: PLoS One. 2017 Sep 18;12(9):e0184721. doi: 10.1371/journal.pone.0184721 (PMC5602537; doi:10.1371/journal.pone.0184721)
Supplement: S1 Appendix — (PDF) [file pone.0184721.s006.pdf]

SUPPLEMENTARY INFORMATION

Comprehensive metabolic characterization of serum osteocalcin action in a large non-diabetic sample

Entenmann L, Pietzner M, Artati A, Hannemann A, Henning AK, Kastenmüller G, Völzke H, Nauck M, Adamski J, Wallaschofski H, Friedrich N

Supplementary Methods

<u>Metabolomics Measurements</u>

Non-targeted metabolomics analysis for metabolic profiling was conducted at the Genome Analysis Center, Helmholtz Zentrum München. Two separate LC-MS/MS analytical methods mere used as previously published, i.e. in positive and in negative ionization modes, were used to detect a broad metabolite panel 1. In this study, samples were divided into two sets according to the biological matrices of the samples, i.e. plasma and urine. On the day of extraction, samples were thawed on ice. A 100µL of the sample were pipetted into a 2mL 96-well plate. In addition to study samples, a human pooled reference plasma sample (Seralab, West Sussex, United Kingdom) and another pooled reference matrix of each sample set (Seralab, West Sussex, United Kingdom) were extracted and placed in 1 and 6 wells, respectively, of the 96-well plate. These samples served as technical replicates throughout the data set to assess process variability. Beside those samples, 100µL of water was extracted as samples and placed in 6 wells of the 96-well plate to serve as process blanks. Protein was precipitated and the metabolites were extracted with 475µL methanol, containing four recovery standards to monitor the extraction efficiency. After centrifugation, the supernatant was split into 4 aliquots of 100µL each onto two 96-well microplates. The first 2 aliquots were used for LC-MS/MS analysis in positive and negative electrospray ionization mode. Two further aliquots were kept as a reserve. The extracts were dried on a TurboVap 96 (Zymark, Sotax, Lörrach, Germany). Prior to LC-MS/MS in positive ion mode, the samples were reconstituted with 0.1% formic acid (50µl for plasma, 100µl for urine). Whereas samples analyzed in negative ion mode were reconstituted with 6.5mM ammonium bicarbonate (50µl for plasma, 100µl for urine), pH 8.0. Reconstitution solvents for both ionization modes contained internal standards that allowed monitoring of instrument performance and also served as retention reference markers. To minimize human error, liquid handling was performed on a Hamilton Microlab STAR robot (Hamilton Bonaduz AG, Bonaduz, Switzerland). LC-MS/MS analysis was performed on a linear ion trap LTQ XL

mass spectrometer (Thermo Fisher Scientific GmbH, Dreieich, Germany) coupled with a Waters Acquity UPLC system (Waters GmbH, Eschborn, Germany). Two separate columns (2.1 x 100 mm Waters BEH C18, 1.7 µm particle-size) were used either for acidic (solvent A: 0.1% formic acid in water, solvent B: 0.1% formic acid in methanol) and or for basic (A: 6.5mM ammonium bicarbonate, pH 8.0, B: 6.5mM ammonium bicarbonate in 95% methanol) mobile phase conditions, optimized for positive and negative electrospray ionization, respectively. After injection of the sample extracts, the columns were developed in a gradient of 99.5% A to 98% B over an 11 min run time at 350µLl/min flow rate. The eluent flow was directly run through the ESI source of the LTQ XL mass spectrometer. The mass spectrometer analysis alternated between MS and data-dependent MS/MS scans using dynamic exclusion and the scan range was from 80-1000 m/z. Metabolites were identified by Metabolon, Inc. from the LC-MS/MS data by automated multiparametric comparison with a proprietary library, containing retention times, m/z ratios, and related adduct/ fragment spectra2. Identification criteria for the detected metabolites are described in Evans et al.1. Quality control methods and normalization of metabolite levels are explained in detail in the supplement.

Metabolomics Measurements: Quality Control and Normalization of Metabolite Levels

To correct for daily variations of platform performance, the raw ion count of each metabolite was rescaled by the respective median value of the run day. Valid estimation of the median was ensured by keeping only metabolites with at least three measured values on more than the half of the run days. This procedure resulted in 475 and 558 metabolites for plasma and urine, respectively, available for the present analysis. 263 metabolites were measured in both bio fluids. We chose probabilistic quotient normalization (PQN) 3 to account for diurnal variation of urine samples, since this procedure was shown to be superior to the common creatinine scaling. For this purpose we calculated a mean-pseudo-spectrum depending on metabolites with measurements for all participants (131 urine metabolites). Subsequently, we calculated a dilution factor as the median quotient between the reference spectrum and each sample. Of note, urine creatinine and the estimated dilution factor were highly correlated ($r=0.91$, $p<0.001$) within the present study sample. Afterwards all metabolite levels were log2-transformed. Separately for plasma and urine samples we performed multivariate outlier detection using an algorithm proposed by Filzmoser et al. 4 as implemented in the pcout function within the R package mvoutlier. The algorithm provides an outlier score for each sample based on a weighted combination of location and scatter estimations using principle

component analysis and the Mahalanobis distance on a robustly scaled data matrix. The default parameters were used for the identification process, except the critical value for the location outliers was set to 4, as it corresponds to a 4 SD exclusion criteria. The minimum score was used as cut-off for outlier identification. As a result 13 and 8 samples from plasma and urine were excluded, respectively.

References

1 Evans, A. M., DeHaven, C. D., Barrett, T., Mitchell, M. & Milgram, E. Integrated, nontargeted ultrahigh performance liquid chromatography/electrospray ionization tandem mass spectrometry platform for the identification and relative quantification of the small-molecule complement of biological systems. Analytical chemistry 81, 6656-6667, doi:10.1021/ac901536h (2009).

2 Lawton, K. A. et al. Analysis of the adult human plasma metabolome. Pharmacogenomics 9, 383-397, doi:10.2217/14622416.9.4.383 (2008).

3 Dieterle, F., Ross, A., Schlotterbeck, G. & Senn, H. Probabilistic quotient normalization as robust method to account for dilution of complex biological mixtures. Application in 1H NMR metabonomics. Analytical chemistry 78, 4281-4290, doi:10.1021/ac051632c (2006).

4 Filzmoser, P., Maronna, R. & Werner, M. Outlier identification in high dimensions. Computational Statistics and Data Analysis 52, 1694-1711 (2008).
